# Comparison of cytotoxicities and anti-allergic effects of topical ocular dual-action anti-allergic agents

**DOI:** 10.1186/s12886-019-1228-5

**Published:** 2019-11-08

**Authors:** Sung Il Kim, Choul Yong Park, Gladys Fordjuor, Jong Heon Lee, Jong Soo Lee, Ji Eun Lee

**Affiliations:** 1 Department of Ophthalmology, School of Medicine, Pusan National University, Mulgumup, Yangsan, 50612 Gyeongnam Province Republic of South Korea; 2 Research Institute for Convergence of Biomedical Science and Technology, Pusan National University Yangsan Hospital, Yangsan, South Korea; 3 Department of Ophthalmology, Dongguk University Ilsan Hospital, Dongguk University College of Medicine, Goyang, South Korea; 4 Ophthalmology Unit, Department of Surgery, Korle-Bu Teaching Hospital, Accra, Ghana; 5 Busan Medical Center, Busan, South Korea

**Keywords:** Allergic conjunctivitis, Cytotoxicity, Topical anti-allergic agents

## Abstract

**Background:**

To investigate the cytotoxicities of the topical ocular dual-action anti-allergic agents (alcaftadine 0.25%, bepotastine besilate 1.5%, and olopatadine HCL 0.1%) on human corneal epithelial cells (HCECs) and their anti-allergic effects on cultured conjunctival epithelial cells.

**Methods:**

A Methylthiazolyltetrazolium(MTT)-based calorimetric assay was used to assess cytotoxicities using HCECs at concentrations of 10, 20 or 30% for exposure durations of 30 min, 1 h, 2 h, 12 h or 24 h. Cellular morphologies were evaluated by inverted phase-contrast and electron microscopy. Wound widths were measured 2 h, 18 h, or 24 h after confluent HCECs monolayers were scratched. Realtime PCR was used to quantify anti-allergic effects on cultured human conjunctival cells, in which allergic reactions were induced by treating them with *Aspergillus* antigen.

**Results:**

Cell viabilities decreased in time- and concentration-dependent manners. Cells were detached from dishes and showed microvilli loss, cytoplasmic vacuoles, and nuclear condensation when exposed to antiallergic agents; alcaftadine was found to be least cytotoxic. Alcaftadine treated HCECs monolayers showed the best wound healing followed by bepotastine and olopatadine (*p* < 0.0001). All agents significantly reduced the gene expressions of allergic cytokines (IL-5, IL-25, eotaxin, thymus and activation-regulated chemokine, and thymic stromal lymphopoietin) and alcaftadine had the greatest effect (*p* < 0.0001 in all cases).

**Conclusions:**

Alcaftadine seems to have less side effects and better therapeutic effects than the other two anti-allergic agents tested. It may be more beneficial to use less toxic agents for patients with ocular surface risk factors or presumed symptoms of toxicity.

## Background

The prevalence rates of allergic diseases have been increasing due to hereditary factors, environmental pollution, increased allergen levels, and changes in life patterns including dietary [1, 2]. Approximately 6–30% of individuals are suffering from allergic conjunctivitis (AC) and 30~70% of them accompany other allergic diseases [3, 4]. Even though AC is not a life-threatening disease, its chronic, recurrent tendencies influence the quality of patient’s life considerably [5].

The fundamental treatment for AC is to avoid allergens that cause hypersensitive reactions as other allergic diseases. However, it is difficult not only to identify the causative allergens accurately but also to avoid a known allergen completely if they are easily encountered in daily life. For these reasons, pharmacotherapy has been used to provide symptom relief and treatment in AC.

The clinical manifestations of AC such as itching, hyperemia, chemosis, and eyelid swelling are the result of mast cell degranulation and the release of inflammatory chemical mediators (especially histamine), which are initiated by crosslinking between permeated allergen and sensitized IgE on mast cell surface [6]. Therefore, these pathologic immune reactions have been considered as main targets for pharmacotherapy, and can be controlled by antihistamine agents and mast cell stabilizers. Olopatadine was the first approved dual-action topical agent and other two dual-action agents have been developed and become general trend to treat AC. Although dual-action agents reduce dosage and frequency due to its rapid onset and long lasting therapeutic effect, long period of use can damage ocular surface cells [7–9]. An impaired epithelial barrier may allow allergens to infiltrate easily and exacerbate the disease. Therefore, reliable safety as well as therapeutic effects are required for anti-allergic agents.

We chose three topical ocular dual-action anti-allergic agents for this study; alcaftadine 0.25% (Lastacaft®, Allergan, Inc., Irvine, CA, USA) and bepotastine besilate 1.5% (Talion®, Dong-A ST, Seoul, Korea), which were introduced recently, and olopatadine HCL 0.1% (Pataday®, Alcon, Fribourg, Switzerland), a traditionally and widely used agent. The aim of this study was to investigate the cytotoxicities of these agents on cultured human corneal epithelial cells and their anti-allergic effects on cultured human conjunctival epithelial cells in vitro.

## Methods

### Cell lines

This study was performed according to the tenets of the Declaration of Helsinki. The SV-40-transfected human corneal epithelial cell line (HCE-T) was obtained from the American Type Culture Collection (ATCC-CRL-11515; Manassas, VA, USA), and was grown to 80% confluency in keratinocyte serum-free medium (KSFM) containing 0.05 mg/ml bovine pituitary extract and 5 ng/mL epidermal growth factor in collagen-coated plates. Before treatment, the cells underwent epidermal growth factor starvation overnight, as previously described [10].

The Wong Kilbourne derivative of the Chang conjunctival epithelial cell line (WKD; clone 1–5c-4, ATCC, Manassas, VA) was cultured under standard conditions (moist atmosphere, 5% CO_2_, 37 °C) in Dulbecco’s minimum essential medium (DMEM, 340 ± 20mOsM) supplemented with 10% fetal bovine serum (FBS), 1% glutamine (200 mM stock solution), 1% penicillin (10,000 units/ml), and 1% streptomycin (10,000μg/ml) for 24 h to reach confluence before challenges [11].

### Methylthiazolyltetrazolium (MTT) assay

The viabilities of human corneal epithelial cells (HCECs) were measured using a MTT assay (3-[4,5-dimethylthiazol-2-yl]-2,5-diphenyl tetrazolium bromide; Sigma, St. Louis, MO, USA). Cultured cells (100 μl; 5 × 10^4^ cell/ml) were seeded in 96-well tissue-culture plates and incubated at 37 °C in 5% CO_2_ for 24 to 48 h until cultures were subconfluent. Alcaftadine, bepotastine, or olopatadine (100 μl diluted 10, 20%, or 30%) were added and incubated for 0.5, 1, 2, 12 or 24 h. DMEM (100 μl) was added to controls. After drug exposure, plates were washed three times with PBS to remove the drugs. Cell viabilities were evaluated after incubating for 24 h, and MTT was then added to each well. Samples were incubated in the dark for 4 h at 37 °C, and media were then removed. The formazan reaction product was dissolved by adding 150 μl dimethyl sulfoxide (Sigma, St. Louis, MO, USA), and absorbances were measured on an automatic plate reader (Molecular Devices, Sunnyvale, CA, USA) at 570 nm. The experiment was repeated 5 times.

### Morphologic assay

HCECs were exposed to three anti-allergic agents at a concentration of 10% for 24 h and photographed under an inverted phase-contrast light microscope. For transmission electron microscopy, cells that had been grown to confluence in 24-well plates were incubated in DMEM containing 10% concentrations of the three anti-allergic agents or phosphate buffer (control) for 4 or 8 h under 5% CO_2_ at 37 °C. After rinsing with PBS, cells were incubated at 37 °C for 24 h, fixed with 2.5% glutaraldehyde in 0.1 mol/L phosphate buffer (pH 7.4) for 12 h and postfixed with 0.1% osmium tetroxide for 2 h. After rinsing with 0.1 mol/L of a phosphate buffer and dehydrating in a graded ethanol series, specimens were embedded in an Epon 812 mixture. Ultrathin sections (60~80 nm) were then stained with uranyl acetate and lead citrate, and examined under a transmission electron microscope (JEOL1200EX: Jeol Ltd., Tokyo, Japan.)

### Scratch wound healing assay

A scratch-wound assay was used to compare the effects of alcaftadine, bepotastine and olopatadine on corneal epithelial wound healing. HCECs were cultured to confluent monolayers on eight well chamber slides coated with collagen I (10 mg/cm^2^; Auspep, Parkville, VIC, Australia) and then scratched with a 100 μl pipette tip. Cells were then washed with fresh medium to remove detached cells and incubated in medium in the presence 10% concentrations of the three anti-allergic agents for 2, 18, or 24 h. To ensure that wounds in similar areas were compared, multiple positioning marks were made at the center of denuded surfaces with a needle, and mean distances between wound edges were measured. Twenty-four hours after wounding, monolayers were fixed, and wound areas in marked fields of view were imaged. Mean distances between original and migrated wound edges of three separate samples per treatment were determined using an image analysis system (Image J 1.33o;available by ftp at zippy.nimh.nih.gov/ or at http://rsb.info.nih.gov/nih-imageJ; developed by Wayne Rasband, National Institute of Health, Bethesda, MD, USA), and percentage wound closures in response to the three anti-allergic agents were compared. The experiment was repeated 5 times.

### Analysis of electrolyte compositions, pH values, and osmolarities of the eye solutions

The electrolyte compositions of the three anti-allergic agents were assessed using a LX-20 (Beckman Coulter, Fullerton, CA, USA). pH and osmolarity were measured using a Metrohm 780 (Metrohm, Zofingen, Switzerland) and a Micro-Sample Osmometer (Fiske Associate, Norwood, MA, USA), respectively.

### Conjunctival provocation test (CPT)

Cultured conjunctival epithelial cells were seeded with or without 10% concentrations of anti-allergic agents and incubated 2 h at 37 °C under 5% CO_2_. Conjunctival cells were subsequently treated with or without 1 mg/ml *Aspergillus fumigatus* allergen extract (Jubilant Hollister-Stier, Kirkland, Quebec, Canada) for 1 h. Cells were then collected, lysed, and treated with Tri-RNA reagent (Favorgen, Taiwan) to extract mRNA, according to the manufacturer’s instructions. Total extracted RNA was used to generate cDNA using oligo-dT, dNTP, RNasin® ribonuclease Inhibitor, and M-MLV reverse Transcriptase (Promega, Madison, Wisconsin, USA). To quantify cytokine gene expression, cDNA samples were amplified in AMPOGENE® qPCR Green Mix Lo-ROX (Enzo Life Sciences, Farmingdale, NY, USA). The primer pairs used for RT-PCR are shown in Table 1. The experiment was repeated 5 times.

**Table 1:** List of PCR primer sequences

Cytokines	Primer sequence (5′-3′)
GAPDH	F: AAT CCC ATC ACC ATC TTC CA
	R: TGG ACT CCA CGA CGT ACT CA
Eotaxin	F: TCT GTG GTC ATC CCC TCT CC
	R: TTG GCG TCC AGG TTC TTC AT
IL-5	F: TAC GTG TAT GCC ATC CCC AC
	R: CCC CCT TGC ACA GTT TGA CT
IL-25	F: GCT GCT CTA CCA CAA CCA GA
	R: GTG GTT GTA CAC CTG GCT CC
TSLP	F: TGG GTG TCC ACG TAT GTT CC
	R: ACT CGG TAC TTT TGG TCC CAC
TARC	F: TGT TCG GAC CCC AAC AAC AA
	R: TCA CTG TGG CTC TTC TTC GTC

*F* Forward, *GAPDH* Glyceraldehyde-3-phosphate dehydrogenase, *IL* interleukin, *R* Reverse, *TARC* thymus and activation-regulated chemokine, *TSLP* thymic stromal lymphopoietin

### Statistical analysis

Statistical analysis significance was determined by ANOVA followed by Tukey’s post hoc analysis (Prism; GraphPad Software, La Jolla, CA, USA). Statistical significance was accepted for *p* values < 0.05.

## Results

**Fig. 1:**
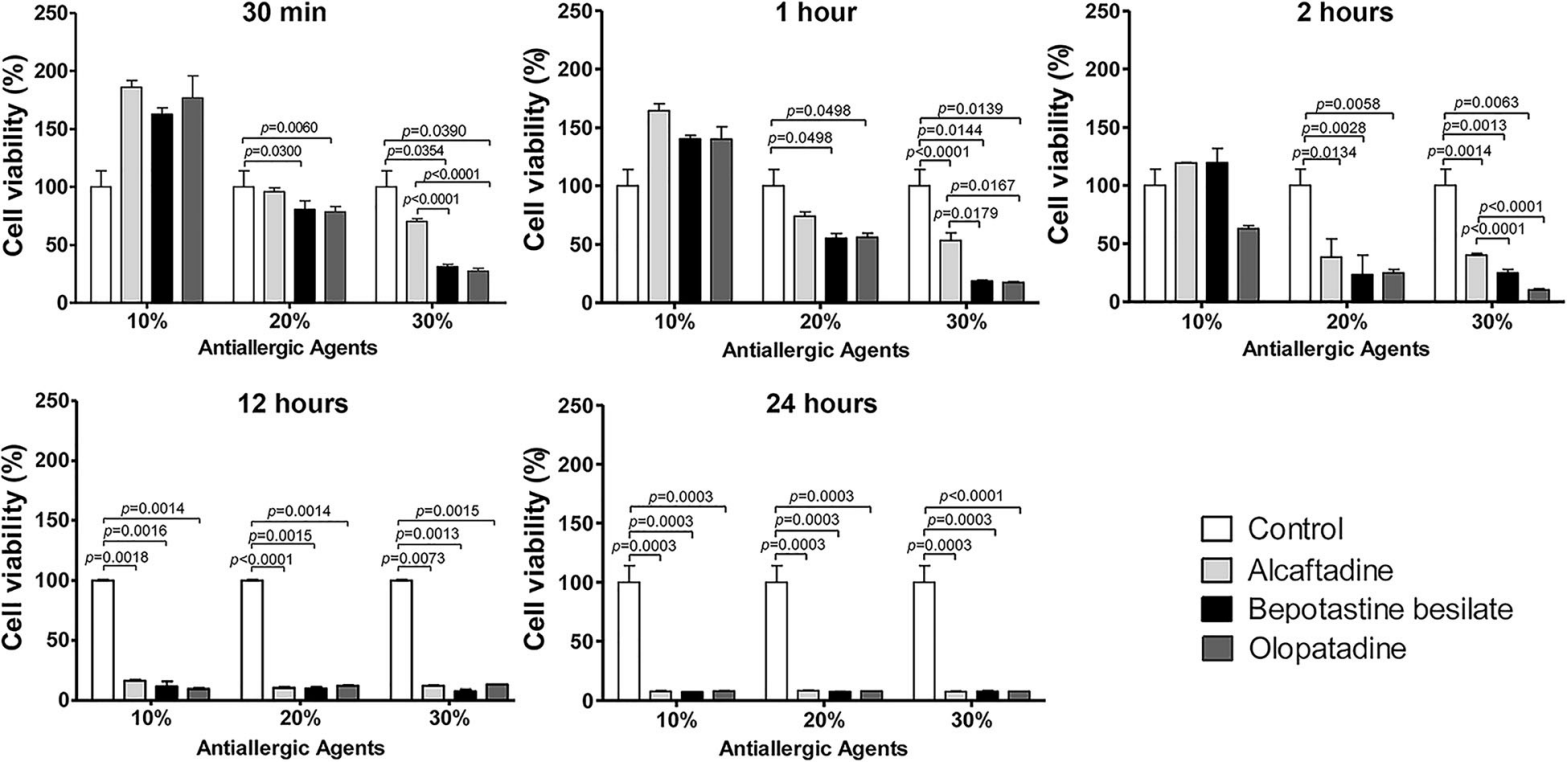
The viabilities of human corneal epithelial cells (HCECs) evaluated by MTT assay. Cell viability was found to be time and concentration dependent and to be significantly reduced after 12 h exposure to all antiallergic agents. At a concentration of 30%, bepotastine and olopatadine treated HCECs were significantly less viable than alcaftadine treated HCECs at exposure times up to 2 h. Survival rates are provided as means ± SDs

HCECs viabilities after exposure to the three anti-allergic agents at different dilutions and exposure times are shown in Fig. 1. At a concentration of 30%, viability decline in alcaftadine was the smallest compared with the control and alcaftdine showed significantly higher viability than bepotastine or olopatadine at exposure times up to 2 h. At a concentration of 20%, viabilities in bepotastine and olopatadine were significantly lower than in the control after 30 min, whereas alcaftadine had no significant effect at exposure times up to 2 h. At exposure times of ≥12 h, the comparisons between agents were meaningless because viabilities were extremely low.

**Fig. 2:**
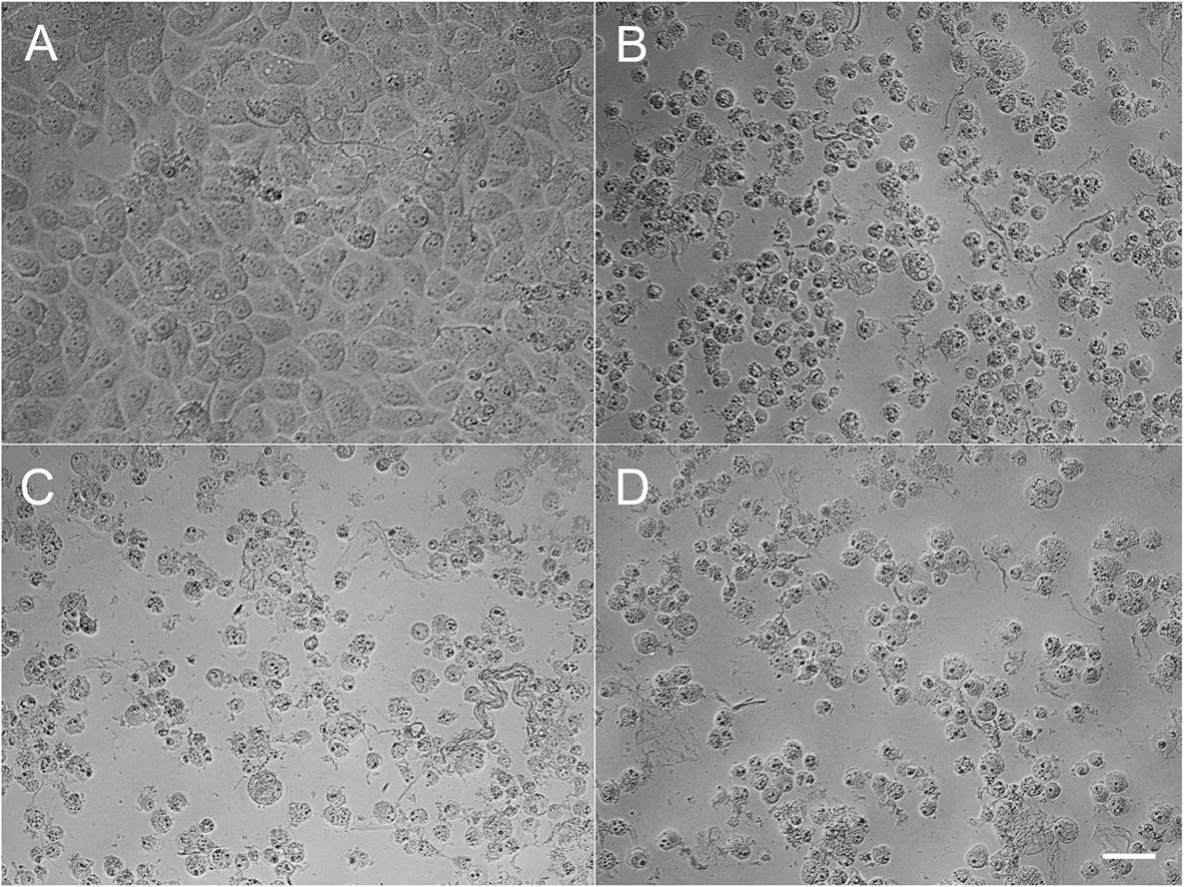
Inverted phase contrast micrographs of human corneal epithelial cells (HCECs) exposed to 10% antiallergic agents (bar length 50 μm, original magnification × 200). Many epithelial cells were visible in control culture media (**a**). HCECs were less detached from dishes after treatment with alcaftadine (**b**) than after treatment with bepotastine (**c**) or olopatadine (**d**)

Phase-contrast microscopy revealed many epithelial cells were densely arrayed in control culture media (Fig. 2a), but in the presence of either of the three agents HCECs progressively detached from dishes (Fig. 2b-d), although alcaftadine exposed cells were less detached and more densely arrayed than cells exposed to bepotastine or olopatadine (Fig. 2b).

**Fig. 3:**
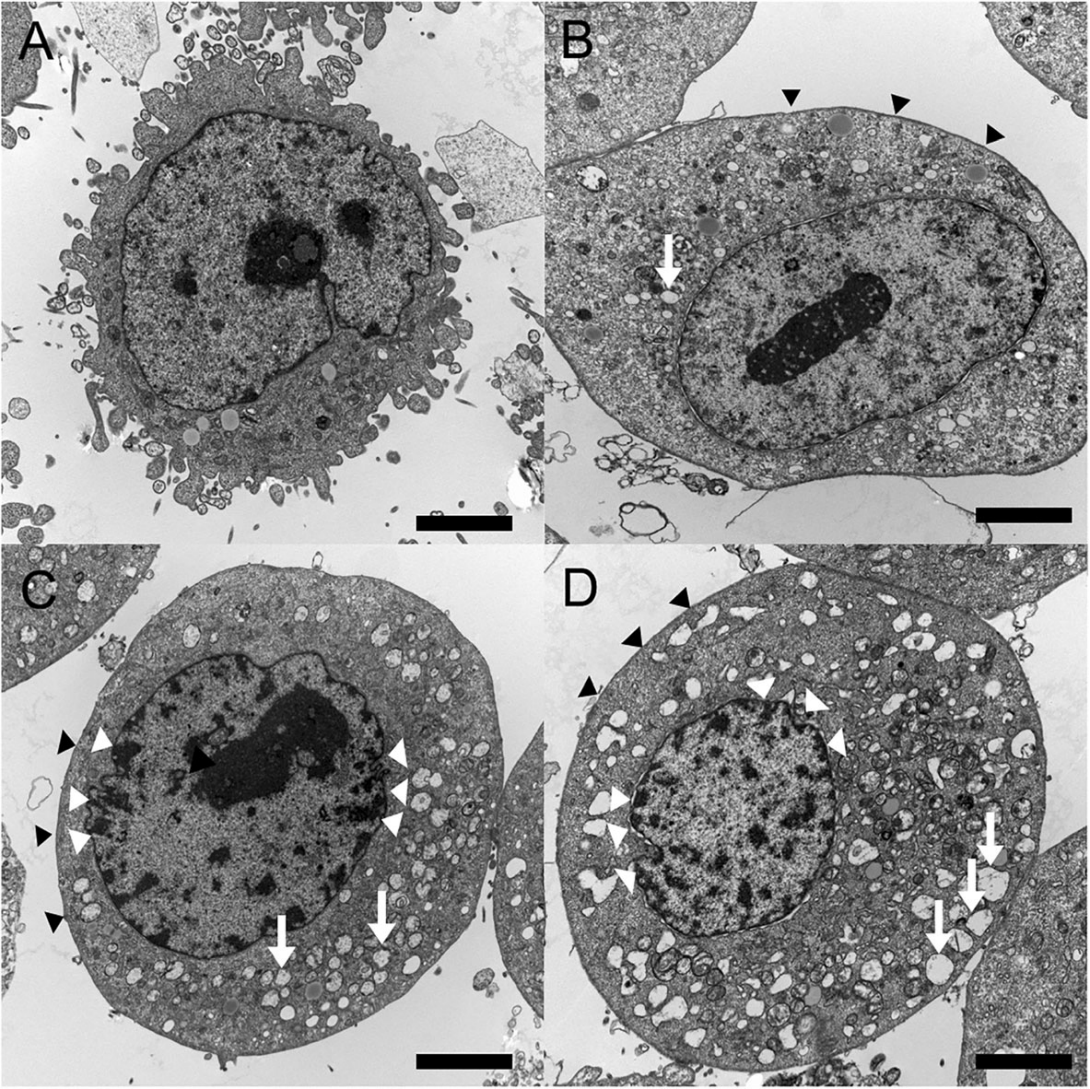
Transmission electron micrograph of human corneal epithelial cells (HCEC) (bar length 2 μm, original magnification × 3000–4000). HCECs were exposed to culture media (**a**), and 10% diluted solutions of antiallergic agents; alcaftadine (**b**), bepotastine (**c**), and olopatadine (**d**). Normal corneal epithelial cells (**a**) showed microvilli, homogenous cytoplasm and intact cells and nuclear membranes. Antiallergic agents exposed cells (**b**, **c** and **d**) exhibited damage to plasma membranes, loss of microvilli (black arrowheads), increased and enlarged vacuoles (white arrows), and nuclear chromatin condensation (white arrowheads). Olopatadine treated cells showed more and larger vacuoles and condensed nuclear remnants along nuclear peripheries

Electron microscopy showed that HCECs exposed to alcaftadine, bepotastine, or olopatadine demonstrated more cytoplasmic bleb formation and loss of microvilli (Fig. 3b-d) than control cells (Fig. 3a). Whereas cells exposed to alcaftadine showed minimal changes, cells exposed to bepotastine or olopatadine showed more and larger cytoplasmic vacuoles and nuclear chromatin condensation along nuclear peripheries (Fig. 3c, d).

**Fig. 4:**
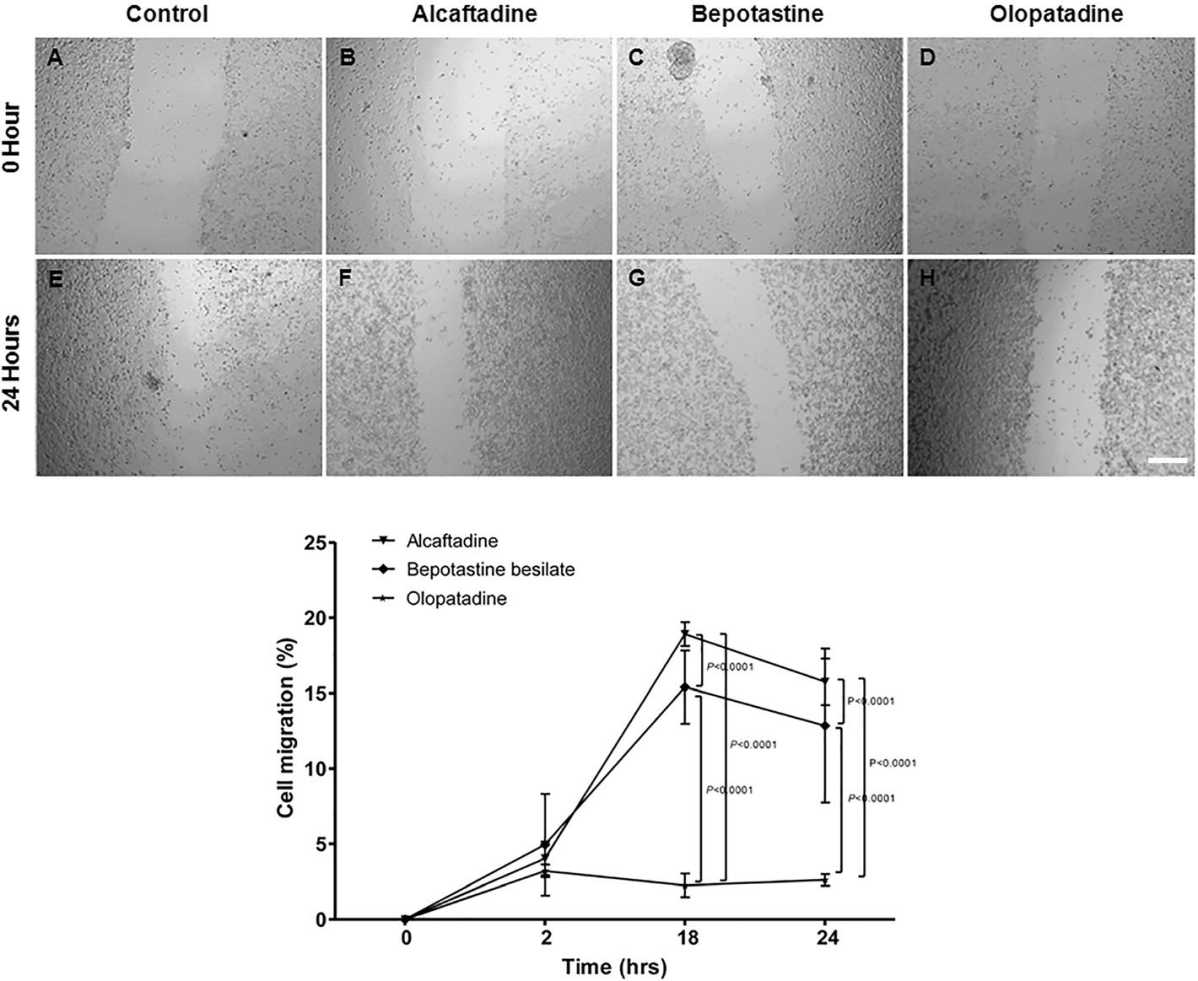
The closure of human corneal epithelial cells (HCECs) wounds in response to 10% antiallergic agents (bar length 200 μm, original magnification × 4). Migration was assessed after 2, 18 or 24 h after scratching confluent HCECs in the presence or absence of antiallergic agents. Micrographs show wound widths immediately after and 24 h after wounding in the absence of any agent (**a**, **e**) or in the presence of 10% alcaftadine (**b**, **f**), bepotastine (**c**, **g**) or olopatadine (**d**, **h**). The effects of antiallergic agents are expressed as percentage reductions in average wound widths. Results are expressed as means ± SDs of percentage wound widths (defined as average widths at 10 positions). Wound widths were significantly narrower for cells exposed to alcaftadine than for cells exposed to bepotastine or olopatadine

Eighteen and 24 h after scratching HCECs monolayers, alcaftadine exposed HCECs exhibited significantly better wound healing than bepotastine or olopatadine exposed cells (*p* < 0.0001). Olopatadine treated wounds showed almost no change (Fig. 4).

The measured values of electrolytes, pH, and osmolarity, and preservative are shown in Table 2. The concentrations of Na^+^ and Cl^−^ in bepotastine were lower and in olopatadine were higher than ideal ranges, whereas the concentration of K^+^ in all three agents was lower than the ideal range; bepotastine had the lowest value. Alcaftadine had the highest Cl^−^ level and was more acidic than the other agents. The osmolarities of all agents were within normal limits. Benzalkonium chloride (BAC) was the preservative used in all agents and its concentration in olopatadine was twice as high as in alcaftadine or bepotastine.

**Table 2:** Electrolyte compositions, pH values, osmolalities, and preservative contents of the three anti-allergic agents

Parameters	Anti-allergic agents			Ideal range [12–14]
	Alcaftadine 0.25%	Bepostatine besilate 1.5%	Olopatadine HCL 0.1%	
Na ^+^ (mEq/L)	151.7	135.9	177	142.0–152.7
K^+^ (mEq/L)	0.93	0.75	1.46	4.3–4.6
Cl^−^ (mEq/L)	145.0	99.3	118.1	104.0–117.4
Osmolarity (mOsm/kg)	292	275–350	296	260–320
Preservative (BAC, %)	0.005	0.005	0.01	< 0.025
pH	7.2	6.5–7.1	7.3	7.0–7.7

*BAC* benzalkonium chloride

**Fig. 5:**
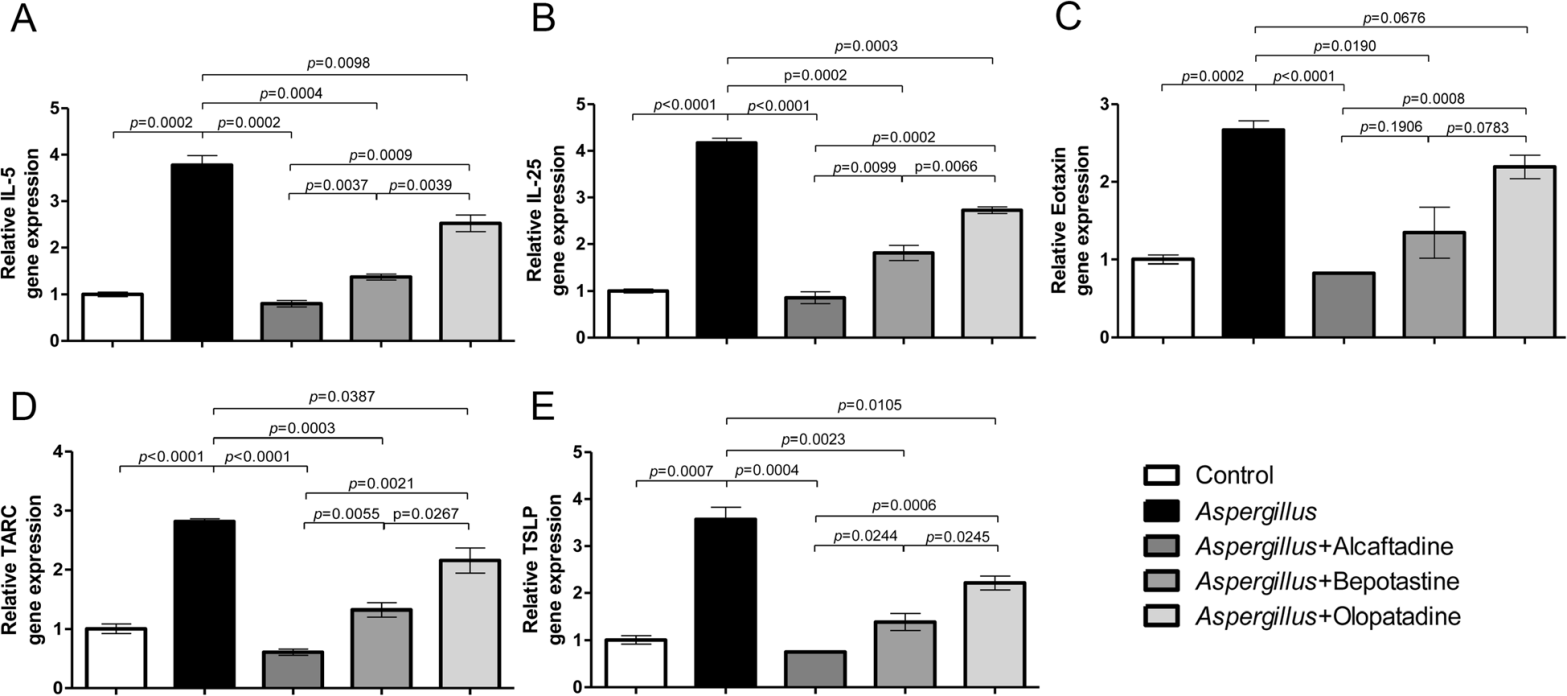
Quantitative real time PCR analyses of the mRNA expression of allergic cytokines in conjunctival cells. Levels of *interleukin(IL)-5* (**a**), *IL-25* (**b**), *eotaxin* (**c**), *TARC* (thymus and activation-regulated chemokine) (**d**), and *TSLP* (thymic stromal lymphopoietin) (**e**) are shown. Expression levels of all 5 cytokines were lowest for alcaftadine. Results are presented as means ± SDs

All three anti-allergic agents significantly reduced the gene expressions of allergic cytokines induced by *Aspergillus* allergen provocation, except eotaxin induction by olopatadine in conjunctival cells. Alcaftadine had the greatest effect on the reduction of all cytokine gene expressions examined (Fig. 5).

## Discussion

Dual-action anti-allergic agents are widely and commonly used for AC which is not severe as demanding steroid or immune modulators. The aim of the present study was to compare the cytotoxicities and anti-allergic effects of the commercially available topical dual-action anti-allergic agents; alcaftadine 0.25%, bepotastine besilate 1.5%, and olopatadine HCL 0.1%.

MTT assay revealed that cell viabilities decreased with exposure time and agent concentration. Bepotastine and olopatadine induced significantly lower viabilities than the control even after 30 min at concentrations of 20 and 30%, whereas alcaftadine induced significantly lower viabilities after 2 h at a concentration of 20% and 1 h at a concentration of 30%. In addition, cell viability in the presence of alcaftadine was significantly higher than in the presence of bepostatine or olopatadine at a concentration of 30%. However, after treatment for more than 12 h including 24, 48 and 72 h (data not shown for 48 and 72 h), all agents proved toxic to HCECs, which agrees with other studies [15, 16].

A comparative study of olopatadine and alcaftadine on murine conjunctival epithelial cells concluded that alcaftadine had a protective effect on epithelial tight junction protein expression [17]. This property could explain why in our study alcaftadine treated cells detached to a lesser extent than bepotastine or olopatadine treated cells. Cytoplasmic blebbing, chromatin clumping and margination, and loss of microvilli are the evidences of cellular damage caused by chemical, mechanical or hypoxic injury [18]. Alcaftadine exposure resulted in less severe cellular changes than bepotastine or olopatadine.

Abnormal electrolyte composition, pH, and osmolarity can damage cellular functions [19]. In the present study, most measured electrolyte values were beyond ideal ranges. Abnormal electrolyte composition not only augments agent toxicity, due to changes in cell membrane permeabilities, but can also cause other types of cell damage. On the other hand, osmolarity and pH of all agents may not affect the toxicity because they were similar and in normal ranges. Preservatives are necessary to prevent ocular infection by prohibiting microorganism proliferation, but they can damage the ocular surface [20–22]. All three agents examined contained BAC, the most commonly used ophthalmic preservative. BAC causes surface-active molecules to bind to cellular epithelium and rapidly intercalate into the bilaminar membrane, and thus, BAC can disrupt the precorneal tear film and damage the ocular surface [16, 23–25]. BAC concentrations in topical ocular solutions typically range between 0.004 and 0.025% [20]. Recently, it was reported that even at the lowest concentration tested, 0.001%, BAC caused significant loss of cellular metabolic activity at exposure times as short as 1 min [26]. Although we found all three agents had BAC levels in the recommended concentration range, olopatadine had twice as much BAC than alcaftadine or bepotastine. This higher level of BAC could partly explain the lower viability of olopatadine treated HCECs. These results are consistent with those of previous studies, in which cell viabilities were found to be affected more by anti-allergic drugs containing BAC [16, 21, 23]. Similarly, BAC-containing drugs used to treat other chronic ocular pathologies, like glaucoma, have been reported to be more cytotoxic than preservative free preparations [24, 25].

Damage, abrasions, or wounding of epithelial cells are caused by various insults including ophthalmic agents which can impair healing [27]. Healing involves a series of events that includes the proliferation and migration of cells to seal wounds [28, 29]. In the present study, alcaftadine treated cell layers showed the best wound healing followed by bepotastine treated cell layers. Olopatadine treated cell layers showed least wound healing, in fact, wound gaps did not almost change. Interestingly olopatadine has been reported to inhibit monocyte migration by binding to S100A12 protein, which is involved in inflammation [30]. It is generally considered well-maintained healing capacity helps to minimize harmful effects resulting from a damaged ocular surface barrier.

To the best of our knowledge, this is the first study to use a CPT based on an *Aspergillus* allergen stimulus to investigate the anti-allergic effect of agents on cultured conjunctival epithelial cells in vitro. We measured the gene expression of 5 cytokines related to the allergic reaction cascade, that is, interleukin (IL)-5 for eosinophil activation, IL-25 for Th2 response maintenance, eotaxin for eosinophil recruitment, thymus and activation-regulated chemokine (TARC) for Th2 cell migration, and thymic stromal lymphopoietin (TSLP) for dendritic cell differentiation to prime Th2 cells [31–35]. The results obtained showed all three agents reduced gene expressions. In particular, alcaftadine was superior in terms of attenuating the gene expressions of all five cytokines, the levels of which were similar to those in untreated cells. Furthermore, alcaftadine has a 10 times stronger effect on H1 and H2 receptors than olopatadine and affinity for H4 receptor, which olopatadine does not possess [36–38]. Because Th2 cell-driven allergic response is caused by H1 and H4 receptor activations, these results are reasonable and consistent with those of previous studies [39–41]. The lower cytokine gene expression observed indicated that alcaftadine, bepotastine, and olopatadine have strong anti-allergic effects. However, the CPT has a limitation because it involves the exposure of cultured conjunctival cells to agents before they are sensitized, which thus, differs from real life situations because anti-allergic agents are usually used to already sensitized patients in clinic.

The most obvious limitation of the present study is its in vitro design. However, although in vitro results do not always reflect in vivo effects, we believe that our findings provide a valuable guide with respect to optimal clinical usage. In particular, in patients with decreased tear clearance or lack of sufficient tear amounts, such as the elderly, nasolacrimal duct obstruction, and dry eye syndrome, anti-allergic agents administered to ocular surfaces may cause cytotoxic effects [42–45]. Therefore, if these risk factors are anticipated or symptom suggestive of toxicity is encountered, it might be more beneficial to use a less toxic agent.

## Conclusion

In summary, our in vitro results indicate alcaftadine has less side effects and better therapeutic effects than bepotastine or olopatadine. Although these effects may not correspond with actual response to eye-drops in patients*,* we believe our results provide an advanced guideline to clinicians and better treatment to patients. Well designed in vivo studies should be followed in the future.

## Data Availability

The datasets during and/or analyzed during the current study are available from the corresponding author on reasonable request.

## Abbreviations

AC: Allergic conjunctivitis
ATCC: American type culture collection
BAC: Benzalkonium chloride
CPT: Conjunctival provocation test
DMEM: Dulbecco’s minimum essential medium
FBS: Fetal bovine serum
HCECs: Human corneal epithelial cells
IL: Interleukin
KSFM: Keratinocyte serum-free medium
MTT: Methylthiazolyltetrazolium
TARC: Thymus and activation-regulated chemokine
TSLP: Thymic stromal lymphopoietin
